# Parental Food Choices and Engagement in Raising Children’s Awareness of Sustainable Behaviors in Urban Poland

**DOI:** 10.3390/ijerph18063225

**Published:** 2021-03-20

**Authors:** Ewa Halicka, Joanna Kaczorowska, Krystyna Rejman, Agata Szczebyło

**Affiliations:** Institute of Human Nutrition Sciences, Warsaw University of Life Sciences WULS-SGGW, Nowoursynowska 159c., 02-776 Warsaw, Poland; krystyna_rejman@sggw.edu.pl (K.R.); agata_szczebylo@sggw.edu.pl (A.S.)

**Keywords:** sustainable food consumption, food choices, children, parents, schools, Poland

## Abstract

Promoting sustainable food consumption patterns and understanding factors driving environmentally-friendly food choices is one of the challenges of public health nutrition policies in the 2020s and crucial for the future wellbeing of humans, food systems and the planet as a whole. To assess the impact of sustainability issues on the behaviors of parents living with young school-aged children in Poland a CAWI survey of 1035 adults in urban areas was conducted. A clustering procedure revealed that two of the identified clusters (73% of the sample) rated sustainability factors as important when purchasing food for children but only one of these clusters (29% of the sample) was actively engaged in raising their child’s awareness about sustainable behaviors. The third cluster (27% of the sample) had no intentions to teach their children about food sustainability because of lack of time or distrust in these topics. More than 80% of the sample agreed that parents share a responsibility in teaching children about the links between food, health and environment. Principles of healthy and sustainable diets should be incorporated into public health programmes to empower family members to engage in raising their children’s awareness and adopt more healthy and environmentally-friendly food consumption practices.

## 1. Introduction

Parents play a key role in what their children eat and often act as health promoters, role models and educators in their children’s lives [1]. They are also responsible for providing and choosing food for the household, especially at the first stages of family life cycle. The arrival of children and the responsibility of raising and feeding them was found to be a turning point in many adults’ consumption patterns [2]. In the case of infants feeding practices are usually included in the country’s social, food, health or nutrition policies and are also voluntarily regulated at international levels [3,4]. This is understandable, since diet, including breastfeeding, is an important factor in the programming of health and metabolism in later life and adulthood [5]. In Poland feeding children in nurseries is governed by country regulations, covering the principles of healthy nutrition of children according to current standards of paediatric dietetics. It is recommended to use a model food ration for children aged 2–3 years [6] and product purchase specification [7], and meal safety is ensured by applying Good Manufacturing Practice and Good Hygiene Practice procedures as well as HACCP system [8].

As children reach pre-school age, institutional interest in their development declines although school educational institutions have to control what food is available during school hours. In Poland, feeding is regulated by legal acts, such as the Act on Food and Nutrition Safety [9], the Act on the Education System [10], which obliges local authorities (municipalities) to provide schools with food for children and adolescents, and the Cabinet of Ministers’ Regulation on the “A meal at school and at home” programme [11], which supports the organisation of canteens and eating facilities in schools. In view of the worrying health indicators of school children and adolescents, in order to prevent overweight and obesity and other chronic non-communicable diseases related to improper nutrition, the Minister of Health in the 2016 Regulation [12] defined the requirements for food served in school and the range of food products that can be sold in school canteens, buffets and vending machines. 

The Committee on Human Nutrition of the Polish Academy of Sciences, respecting the provisions of this regulation, postulates the introduction of new standards for planning school menus and the modification of current school meals towards the principles of the “planetary diet” presented by the EAT-Lancet Committee [13]. In the 2019 Position Statement [14], the Committee argues that it is essential to develop good health and pro-environmental habits in schools from an early age. Diets providing local, seasonal and sustainably produced products should be introduced and care should be taken to ensure unlimited access to tap water in schools. Attention needs also to be drawn to the prevention of food waste.

Overall in many countries, including Poland, schools provide a physical, social and educational environment for children and have the ability to shape both physical activity and eating behaviors [15]. A recent Cochrane systematic review of childhood obesity prevention interventions reported that in the primary school-aged population combined dietary and PA interventions in schools had a small positive effect on body mass index [16]. Such intervention programmes implemented in Polish kindergartens and primary schools are most often focused on shaping healthy eating habits. For example, the nationwide programmes “Academy of healthy preschoolers” or “Healthy eating, healthy growing” supports nursery and kindergarten staff and parents in forming healthy eating habits in children aged from 1 to 6 years [17]. Since 2006 the Warsaw local government has been conducting a social campaign “I know what I eat” which includes systematic activities for children, adolescents and their parents, as well as teachers and directors of schools and kindergartens [18]. “Keep Fit” is another example of a programme, implemented in primary schools, which educates children about healthy lifestyles, combining aspects of a healthy diet and high physical activity [19]. Despite these programmes 1/3 of eight-year-olds in Poland feature excessive weight [12] and this problem is most common among children living in cities with over 500 thousand inhabitants (especially when both parents work) and in the rural areas [20]. The increasing prevalence of childhood obesity in countries of all income levels has been recognised as an epidemic and a crisis [21] with multiple individual, public health, and societal consequences, including predictions that it will result in a decline in life expectancy [22]. 

It must be also underlined that socialisation processes, in which young people are taught the skills, behavioral patterns, values and motivations needed to function competently, also in the context of food consumption should involve parents conveying to their children learning outcomes, such as knowledge and attitudes, through a range of behaviors [23]. This is especially true for children before adolescence [24], since for them parents are the primary socialization agents [25]. Their influence regarding food consumption patterns is much greater than that of other role models, such as teachers or classmates [26]. The family eating environment, parental food attitudes, eating behavior and feeding practices are central in the development of children’s eating habits [27], so adults should provide a good model of this behavior for children. Although the role of parents is emphasised, the maternal and paternal influences can be different in the socialisation process of children [28]. Parents interact with children using context-specific practices and strategies to help them achieve specific goals. A number of parental behaviors are strong correlates of child food consumption behavior [29]. Raising and discussing topics linked to food, health and the environmental protection is one of them. Pro-environmental behaviors include eating local food, so the preference for food products originating from one’s own region (called regional ethnocentrism) may be considered a pattern of sustainable consumption, as its predictors are strongly related to the major pillars of sustainable development [30]. According to [31] consumer ethnocentrism is a general tendency acquired during childhood through the process of socialization. 

Support aimed at changing the perception of diet from adequacy in terms of nutrients and energy provision to environmental adequacy needs to be child and family-centered. Sustainable and healthy diets take into account nutrient recommendations while considering environmental, social/cultural and economic sustainability [32]. Making pro-environmental decisions related to food is a key element of responsible food production and consumption which is one of the 17 UN Sustainable Development Goals set to be achieved by 2030 [33]. The 12th SDG is directly linked to Goal 2, which emphasises the importance of achieving food security, improving nutrition and promoting sustainable agriculture. Therefore all people, including children and youth need to learn about environmental damage, greenhouse gas emissions, food system functioning including intensive farming, local, seasonal and organic food as part of their regular school routine [34]. In the framework of the Sustainable School Food Program (financed by the European Commission), seven countries participate in the implementation of an integrated strategy promoting local economy, environmental sustainability and quality in school feeding. Kindergarten and primary school pupils are involved in learning different issues related to healthy and sustainable food systems [35]. Within the 2015 Milan Urban Food Policy Pact, the number of cities that have developed and are implementing sustainable urban food policies, in which children are participants or the target group of many activities, is increasing [36]. European cities which have such a policy include, for example, Milan, Paris, Utrecht, Copenhagen, Edinburgh, Bristol, Gent, and Riga. In Poland’s capital city—Warsaw—work on preparing such an urban policy is nearing completion. The BioCanteens Transfer Network (financed by the European Regional Development Fund) is based on the daily distribution of meals that are 100% organic and mostly composed of local products, the drastic reduction of food waste thereby fully compensating the higher cost of switching to organic products, and the organisation of dedicated educational activities to raise children’s awareness about sustainable food [37].

In Poland some recent initiatives include programmes dedicated to non-food principles of healthy diets, such as those promoting drinking water “Mom, dad, I prefer water!”, especially tap water—“Warsaw Tap Water” [38] or the “EcoMission I don’t Waste” programme of the Federation of Polish Food Banks, which aims to increase environmental awareness of food waste and shape pro-environmental attitudes in primary schools [39]. However sustainable education in schools has to be reinforced in children’s homes. Parental choices exert a significant influence on children’s behaviors and talking about their environmental impact in families with small children can help modify children’s attitudes and behaviors [40]. It has been shown [41] that the behaviors of parents have a significant impact on children’s knowledge of and attitudes towards the environment.

Having in mind the urgent need to accelerate the process of shifting food consumption behaviors towards sustainability we carried out a questionnaire-based survey among Polish adults with young school-aged children. The aim in the presented analysis is to examine, based on the collected data, if parents consider sustainability-linked factors when buying food for their children and to assess the parents’ engagement in raising selected health-food-environment issues—such as buying locally-produced and seasonal food, limiting food waste and plastic water bottle purchases etc.—with their children. In this paper we present the methodology applied in the study, the results of the clustering procedure and discussion of collected data in the wider context of effectively promoting environmentally-friendly food consumption behaviors in families with children. 

## 2. Materials and Methods

### 2.1. Data Collection and Sample

Data on food practices in Polish families with children in the context of healthy and sustainable consumption patterns were collected with the use of Computer Assisted Web Interviews (CAWIs). The original questionnaire, consisting of 21 questions, was tested on a pilot subset of 50 consumers living in cities and parenting at least one child aged 6–8 years. After introducing small editorial changes to increase the clarity of the questions the survey was sent out electronically among the panel. The research company controlled the sample geographical distribution as the panelists sent in their responses in order to include all 16 voivodships (provinces) of Poland. The highest number of respondents were from the śląskie (206), mazowieckie (194) and łódzkie (156) voivodships. In the sampling process a total of 1035 (N) adult members of a research company’s (GfK Polonia) consumer panel (comprising 144.000 households) completed the 25-min long questionnaire. Panelists that met the inclusion criteria—living in a city with more than 50 thousand inhabitants with a child aged 6–8 years-old—for our research were invited on-line to participate in the study and could voluntarily fill out the questionnaire. For participating in studies panelists receive points which can then be exchanged for cash or prizes. 

If there was more than one child from the specified age group in the household, the respondent was asked to consider the youngest of them when answering the survey questions. Quotas, based on that child’s gender and age, were monitored during the 3-week long sampling procedure. 

### 2.2. Measures

This article presents the analysis of data obtained from eight selected survey questions (Q1–Q8 presented in Supplementary materials, Table S1) conducted in IBM SPSS Statistics version 25 (company, city, state abbrev if USA, country).

Q1 and Q2 focused on the key factors that influenced the parent’s choices when purchasing food for children. In the open-ended Q1 parents could list any—with the maximum of three—determinant of food choices. Q2 consisted of a set of 18 predetermined and randomly rotated factors which the respondents were asked to indicate the importance of using a five-point Likert scale anchored at “1” = “unimportant” and “5” = “very important”. A thematic analysis was used to summarise and categorise all the factors that respondents said influenced their food choices.

Questions Q3 and Q4 focused on 12 selected issues linked to food sustainability in the context of health, environment and food security, namely: maintaining health with proper nutrition; buying seasonal food and local products to reduce food transport (food miles); reducing and segregating food waste; limiting the consumption of animal products and of highly processed products; increasing consumption of natural, minimally-processed food; limiting plastic bottle water purchases; sorting food packaging; prevalence of hunger; need to protect the natural environment. Parents indicated the topics that they discuss with their child and the reasons of not engaging in doing so. 

In Q5 and Q6 respondents were asked if they were familiar with the term “sustainable food consumption” and to select its correct definition among four options. They could also give their own proposal. The correct response was deliberately simplified to “when everyday food consumption is carried out to minimize the impact on natural environment” like in our other surveys conducted among Polish city dwellers [42,43].

The remaining two survey questions which were chosen for analysis concerned the parents’ engagement in teaching their child about the links between food practices/diet, the natural environment and health (Q7 and Q8). 

The socio-demographic questions posed in the survey concerned the respondent’s age, gender, level of education and the total number of children in the household. Survey participants also identified their child’s age, gender and type of school she or he attended. Additionally they typified their child’s everyday diet (typical, gluten free, no meat, no animal products, no/limited sweets, no/limited dairy and/or low in carbohydrates) and assessed their child’s body weight (normal, overweight or underweight). Sharing information about the household’s monthly income (per person) was optional.

### 2.3. Data Analysis

A K-means clustering procedure, based on the perceived importance of the 18 pre-defined factors of food choice (included in Q2), was applied to group respondents into consumer segments. The fundamental idea of clustering techniques is that observations (respondents) assigned to a group are as similar as possible (i.e., high intra-class similarity), whereas observations (respondents) belonging to different groups are as dissimilar as possible (i.e., low inter-class similarity). In k-means clustering, each cluster is represented by its center (i.e., centroid) which corresponds to the mean of points assigned to the cluster. The three-cluster solution was chosen and cluster means were statistically contrasted using One-Way ANOVA with Scheffé tests for post hoc comparisons. Descriptive statistics (frequency, means and cross-tabulations) and ANOVA and Pearson’s Chi-square independence tests were used to examine the differences between the three clusters. The details depended on the measurement scale of the variable and a level of *p* ≤ 0.001 was considered significant. 

## 3. Results

### 3.1. Sample Characteristics 

The detailed socio-demographic characteristics of the studied adults (N = 1035) and their child (chosen a subject) are presented in Table 1.

The average age of respondents (hereafter also called parents or caregivers) who participated in the study was 36 years, ranging from 22 to 62. Parents aged 20–29 years constituted 8% of the sample, 30–39 years 70% and 40–49 years—19%. The remaining 3% were 50+ or did not disclose their age. Almost 68% of the respondents were women, 56% held a higher education degree, 37% had completed secondary schools and 7% held a primary level of education. 

More than half (55%) of the study participants had two children living with them in the household, 31% had one child, 14%—three or more children. The age of children was distributed almost equally: 35% six-year-olds, 35% seven-year-olds and 30% eight-year-olds. The share of girls amounted to 52%. A vast majority of children (89%) attended public elementary schools, 4% went to private schools and 7% to preschools. 

Their child’s body weight was assessed subjectively as “normal” by 84% of the parents. Almost 9% of the sample claimed that their child was overweight and 7% described their child as underweight. The diet of 65% of children was “typical”, in 99% cases the child ate animal products incl. meat. A few types of dietary restrictions were named, such as eliminating or limiting sweets, dairy products and carbohydrates. Circa 3% of children followed a gluten free diet. 

Additional information was collected about the engagement of children in the preparation of meals. A vast majority (83%) of schoolchildren often or always participated in the preparation of meals at home, while 17% did so seldom or never. 

In the surveyed group, 35% of respondents indicated low monthly income in their household, 32% medium, and 20% high (surpassing 3001 PLN/person). Circa 13% of the sample refused to give information about the financial situation of their household.

### 3.2. Classifying the Factors That Influence Parental Food Choices 

#### 3.2.1. Respondent-Identified Factors (Q1)

In response to the open-ended question on food choice determinants (Q1), 2448 records were obtained. The thematic analysis of the unprompted answers showed that 31% of them linked to nutrition, mainly to dietary recommendations and composition of purchased products. Parents in detail described that they choose products containing no additives, no salt, no monosodium glutamate, no fructose-glucose syrup, no “empty” calories or that are high in protein, vitamins etc. The second biggest group of factors mentioned by parents (20%) related to the child’s own food preferences and habits. Respondents put emphasis on the fact that their child should like the taste or overall enjoy the product and some of the children were described as “picky eaters”. The third group of factors (16%) was associated with the child’s physical and mental health and wellbeing (incl. weight problems and food allergies). Factors pointing to the parent’s awareness of food sustainability issues, such as local origin, organic farming and seasonality constituted around 12% of the reported factors. The remaining respondent-identified factors were price (11%), quality (5%) and convenience (3%). The 2% of responses that were not categorized and included such answers as: my child eats what we eat, the packaging must be colourful, novelties, products that are recommended by others.

#### 3.2.2. Pre-Specified Factors (Q2) 

The importance of eighteen pre-specified determinants of food choices in the surveyed sample of Polish parental is presented in Table 2. Three of them proved to be most important and were post-hoc classified as “universal” factors. These were: “child’s health” (4.63), “product’s taste” (4.47) and “principles of healthy eating” (4.26). 

Among the five pre-specified factors linked to sustainability issues (“sustainable” or “collective/we”) factors, “symbols certifying the product’s special-quality attributes” were rated highest (4.12). Such certificates—for example Fair Trade—confirm the exceptional features of food products from the sustainability’s environmental, economic or social perspectives. Sustainability labelling of food quality refers to production that respects the environment, has high nutritional value and low level of processing. The product’s “local origin to reduce food transport” (food miles) and “organic production” were also seen as rather important determinants of food choice (3.91). 

The third category of pre-specified factors was called the “individual” (or “me/my household”) factors incl. “price”, “local origin to support Polish producers”, “convenience” and “certified high quality of product”. Such motives considered the respondent’s individual or household perspective and were rated from 3.80 to 3.95 in the total sample. 

In the last category, namely “external” factors (“opinions of others” or “they”) only the “recommendations of health professionals” and “recommendations by research institutes or experts” were seen as rather important determinants of food choices (3.99 and 3.61). The means of the remaining four factors in this category, that is the: “opinions of teachers”, “opinions of the child’s peers” as well as “advertising” and “consumer trends“ were lowest, oscillating around “3”—“neither important, nor unimportant”.

### 3.3. Consumer Segmentation Based on Food Choice Determinants

Statistical analysis of the collected data on the pre-specified factors led to the identification of three consumer clusters (CLs). The biggest segment (CL1) constituted 44% of the total sample (n1 = 458), CL2 comprised 29% (n2 = 304) and CL3—27% (n3 = 273). 

Based on One-Way ANOVA with Scheffé tests, CL1 had relatively higher means on the “universal” and “sustainable” factors (mirroring CL2) and relatively lower means on “individual” and “external” factors (mirroring CL3).

In the case of one of the most important “universal” factors, namely “child’s health” significant differences were observed between all three clusters (Table 2). The two remaining factors in this category did not differ significantly between CL1 and CL2. CL3 means for “product’s taste” and “principles of healthy eating” were statistically lower.

The cluster means of the key “sustainable” factor—that is “symbols certifying the product’s special-quality attributes” were highest in CL2 and CL1 and significantly lower in CL3. For the rest of the “sustainable” factors, the means in the three clusters were all significantly different, highest in CL2 and lowest (rated as “neither important, nor unimportant”) in CL3.

Significant differences were also observed between clusters in the case of all “individual” factors, with the exception of “price”. The importance of this factor did not differ between CL1 and CL3 but was significantly higher in CL2. However; it must be noted that in CL3 “price” was the fourth most important factor and was rated higher than any factors related to food sustainability.

For each of the “external” factors the means in CL1 and CL3 did not differ but those in CL2 were statistically higher. 

Almost without exception CL2 had the highest mean score for all factors—particularly in the “external” category. CL3 had lowest mean score for all factors, except for “taste”, “price” and “opinions of the child’s peers” that did not differ significantly from CL1. 

Socio-demographic characteristics did not significantly differentiate clusters except for three, namely: number of children, household income level and type of school attended by the respondent’s child (Table 3). In CL2 the number of children was statistically lower and the share of households with medium and high income levels was highest (76%). The share of children attending non-public schools was statistically largest in CL3 (6%). 

### 3.4. Discussing Food, Health and Natural Environment at Home 

More than half of the studied sample confirmed discussing ten or more (out of the twelve randomly rotated) topics linking food, health and the natural environment (Q3). The most frequently marked issues were: maintaining health with proper nutrition, sorting waste, limiting food waste and sorting food packaging (Table 4). A slightly smaller part of the surveyed caregivers admitted talking with their child about buying and eating seasonal food (83%) and the necessity to protect the natural environment (79%). Around 72% of the respondents acknowledged discussing hunger in Poland and worldwide, 69% raised the need to increase consumption of natural foods and to reduce the consumption of highly processed foods. The challenge of choosing local foods to limit food transport was raised by 58% of the surveyed group of parents. Less than half of the respondents confirmed discussing the issues of limiting plastic bottle water purchases (44%) and cutting down on animal products, such as meat or eggs (39%). 

Respondents in CL2 were most active in raising all the pre-defined issues linked to sustainable food consumption practices with their child. The percentage of assertive (“yes”) responses ranged from 70 to over 90%. The responses in CL1 and CL2 did not differ, except for the three practices raised least frequently (buying local foods in order to limit transport, limiting plastic bottle water purchases and reducing the consumption of animal foods such as meat or eggs). A statistically smaller share of parents in CL1 discussed these topics in the presence of their children compared to CL2. 

Respondents in CL3 were significantly less interested in all the topics. Less than half of them discussed the prevalence of hunger in Poland and worldwide (49%), increasing the consumption of natural—minimally-processed foods and reducing the consumption of highly-processed foods. An even smaller share (28%) of CL3 raised the issue of buying local foods in order to limit transport. The issues of limiting plastic bottle water purchases and reducing consumption of animal products was brought up by only 19% and 18% of parents in CL3, respectively. 

**Figure 1:**
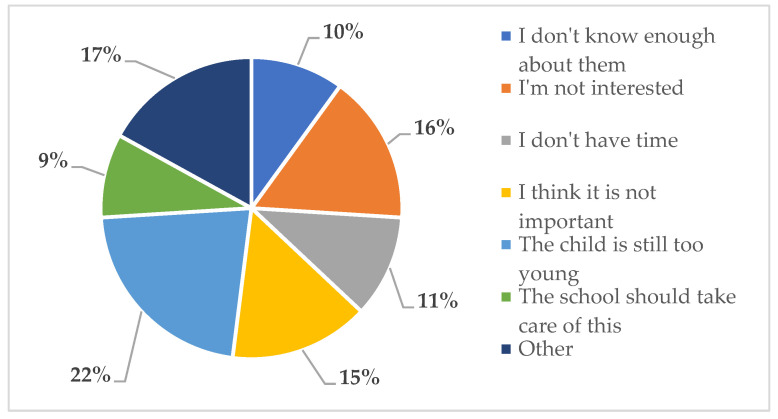
Why do you not include your child in conversations about food sustainability?

The top-one reason (similarly in the total sample and in all clusters) why parents did not involve their child in discussions on topics linking food, environment and health was the fact that the child “is still too young” (22%). This rationale was given most frequently in the case of such topics as: prevalence of hunger in Poland and worldwide, increasing consumption of natural, minimally processed foods and buying local products to reduce food transport (Figure 1).

Circa 17% of the marked responses pointed to “other reasons” for not discussing these issues. Parents explained them with such words as: *I don’t know; it’s hard to tell; it’s nonsense; I am not convinced; I do not see any reason; it’s boring*. This category of answers was selected most frequently in the case of: buying and eating seasonal food, reducing consumption of animal products and limiting plastic bottle water purchases.

Parental lack of interest in the topics specified in Q3 and Q4 and the respondents’ belief that these issues are not important constituted 15–16% (each) of the total number of selected reasons of not including the child in such conversations. These explanations were used mainly in relation to limiting plastic bottle water purchases, sorting food packaging and reducing the consumption of animal products i.e., meat or eggs. 

Lack of time and knowledge constituted around 11% of all the selected reasons for not including the child in conversations linked to food sustainability. Parents admitted to not having enough time to talk about increasing consumption of natural, minimally processed foods, sorting of food packaging and waste segregation. They did not feel confident about discussing especially such issues as: the reduction of animal products in the diet, limiting the plastic bottle water purchases and buying local products to reduce food transport. 

The least frequently selected option to Q4 was that “the school should take care of it” (10% of answers in the total sample)—and was most often chosen in the case of two topics: the need to protect the natural environment and the prevalence of hunger in Poland and worldwide. 

Some statistical differences were found between the three clusters. CL1 parents more often justified the fact that they do not discuss the surveyed topics because they did not consider them important and due to lack of time. CL2 significantly more often admitted that their knowledge is not sufficient. Adults in CL3 and CL2 significantly more often selected the answer “other” and explained the reason in their own words compared to CL1. 

### 3.5. Parents’ Understanding of the Term “Sustainable Food Consumption”

Only 28% of the total sample acknowledged being familiar with the term “sustainable food consumption” and 27% chose the correct answer to Q5 that such consumption implies that “everyday nutrition is carried out in such a way as to minimize its environmental impact”. The majority of respondents (53%) chose the incorrect answer that “food consumption is sustainable when the energy value of consumed food equals the body’s energy expenditure”. Almost 34% believed (also incorrectly) that food consumption is sustainable when “the share of plant and animal products in the food consumed is the same”. The option stating that sustainable food consumption means that “the cost of food is adjusted to the financial capacity of the household” was chosen by 7% of the studied sample. Slightly more than 1% (13 parents) ticked the remaining answer “other”, either repeating that they do not know the term (in most cases) or clarifying that sustainable food consumption implies—in their opinion—low (or zero) food waste. Statistically a smaller share of respondents in CL3 was familiar with the term “sustainable food consumption” but no statistical differences between clusters were found when respondents were asked to indicate its proper meaning. 

### 3.6. Parental Engagement and Responsibility in Increasing the Children’s Understanding of Links between Food, Environment and Health

**Figure 2:**
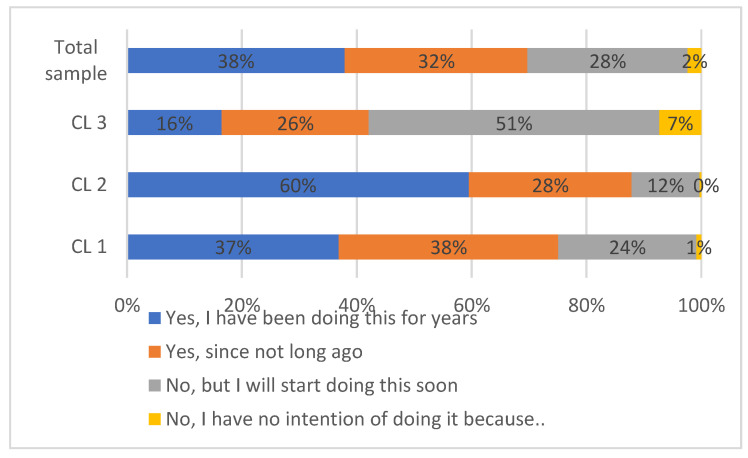
Do you teach your child to pay attention to the natural environment in the context of producing and consuming food?

Despite the low level of awareness of the term “food sustainability” itself, 70% the sample confirmed teaching their child to pay attention to the links between food, health and the natural environment (Q8). Circa half of these respondents had been “doing this for years”. The other 46% of adults who confirmed their involvement admitted “doing this since not long ago” and constituted 32% of the total sample (Figure 2). 

The majority of guardians who admitted not teaching their children about avoiding food waste, the links between nutrition and health and environmental protection intended to start doing so soon. Only 2% of the total sample had no intention of teaching their child because they have: *no interest in these topics, no knowledge, absolutely no time or because it is all nonsense (a story made up by eco-terrorists)*.

Statistically significant differences were observed between clusters (*p*-value 0.000). The share of parents who declared that they taught their child about the links between food and health was 88% in CL2, 75% in CL1 and only 42% in CL3. The majority of parents in CL2 (60%) admitted doing so for years while almost equal shares of parents in CL1 had been doing this for some time or just started doing so. More than half of the parents in CL3 (58%) admitted not teaching their child about these issues. CL3 had the largest share of members who had no intention in becoming engaged in the teaching process (7%) compared to CL2 (0%) and CL1 (1%). 

The collected data also showed that—according to more than 80% of the surveyed parents—the responsibility of teaching children about environmental protection (82%), the link between diet and health (86%) and avoiding food waste (89%) lies with parents. More than half of the respondents indicated that schools are also responsible for such education (52%, 56% and 66%, in CL1–3 respectively). The responsibility of other family members (incl. grandparents) was signaled by 43% respondents in the case of avoiding food waste and 38% on the link between diet and health and environmental protection. Less than 1/3 of all parents pointed to the responsibility of the media (TV, internet, press) to inform schoolchildren about these issues—31% on environmental protection, 26% on the remaining two (each). No statistical differences were observed between clusters.

### 3.7. Cluster Characteristics from the Perspective of Food Sustainability

Analysis of responses to questions Q3–Q8 (see Supplementary Table S1) shed further light on the involvement of the surveyed parents in shaping their child’s understanding of links between food, environment and health. Caregivers in CL2 were genuinely interested and actively involved in discussions on these topics at home however admitted that their knowledge in these fields is insufficient. The statistically largest share (88%) of care-givers in this group, compared to other clusters, declared they taught their child about paying attention to sustainability issues and 60% had been doing this for years. 

Parents in CL1 statistically more often justified their lack of activity in discussing the surveyed topics because they did not consider them important and had not enough time. In CL3 the statistically the biggest share (42%) of parents admitted not teaching their child about these issues and 7% of them had no intentions in becoming involved in the teaching process. This confirms that CL3 can be described as “grey” consumers, who have a rather negative, doubtful approach to sustainability issues, their key priorities in food choice being price and convenience, which are self-oriented (me/my-household) factors. 

## 4. Discussion

The presented data show that the top three factors determining parental food choices among Polish urban inhabitants are: child’s health, the product’s taste and nutrition (healthy eating). Other studies carried out among parents of young children in urban settings also point to similar key food choice motives [27,44]. However it must be noticed that taste preferences of children aged 5–9 years are highly influenced by a biological preference to sweet, salty and sour tastes and an aversion to a bitter taste [45]. Therefore “health” and “taste” as factors of parental food choices may be in contradiction. Indeed, the child’s taste preferences were the caregivers’ main consideration for giving savory snacks and sweets to children, when health-related considerations were connected with fruit snacks [46]. Caregivers often swap healthy options for unhealthy ones, due to other factors such as price, convenience, familiarity, sensory appeal and children’s taste preferences turn out to be decisive factors when choosing food [27,44,47]. Additionally, even when a healthy choice is available, it may not be made if the social norm suggests otherwise [16]. 

Several diverging views on the concepts of sustainable food consumption exist but overall diets that have low environmental impact should be based on a great variety of unprocessed or minimally processed foods, balanced across food groups, while restricting highly processed food and drink products; include wholegrains, legumes, nuts and an abundance and variety of fruits and vegetables; include moderate amounts of eggs, dairy, poultry and fish; and small amounts of red meat [32,48]. Elements of such diets have been included in the revised dietary guidelines of some countries in recent years to promote pro-environmental eating behavior and household food and nutrition management [42].

Sustainable food behavior implies also buying food that meets a credible certified standard [49]. Such certificates are also seen as important in enabling sustainable food consumption patterns and eradicating hunger worldwide. In our research symbols certifying the product’s special-quality attributes were the most important “sustainable” (or “collective”) determinants of food choice, followed by the product’s local origin and organic production. According to research conducted in Spain “Fair trade consumers” tend to have a relatively higher level of education [50]. Other studies found that rural resident consumers have a higher interest in local food issues compared with those in urban areas [51] and that older respondents are more interested in local food compared with younger ones [52]. It must be added that in the European Union organic production implies respecting the rules on organic farming which are designed to promote environment protection, maintain biodiversity and are based on a number of key principles, such as: prohibition of the use of GMOs, forbidding the use of ionising radiation, limiting the use of artificial fertilisers, herbicides and pesticides as well as prohibiting the use of hormones and restricting the use of antibiotics for animal health. The European Green Deal and the 2020 Farm to Fork Strategy comprehensively address the challenges of sustainable food systems in European Union countries. The choice of food labelled with the logo of good accreditation schemes, which are clearly defined, is among the principles of a sustainable and high quality diet [53]. Many studies consider labelling as a means of aggregated communication of environmental and health product features [54,55] and sustainability is considered a competitive advantage within a homogenised market-sector, such as the food sector, and it has been argued that consumers are shaping sustainable market preferences [56].

In the light of our findings parents living in urban Poland appear well intentioned in their motives for selecting food for their children as many respondents purchased foods in line with the child’s desires. This suggests that an increased level of the child’s awareness on sustainability issues (for example on the need to limit purchases of plastic bottles or reduce waste) would further strengthen the impact of “sustainable” factors determining the caregivers’ food choices. 

Consumer segmentation (cluster analysis), as a way to define consumer groups as homogeneously as possible with respect to factors determining food choices is one of the most interesting elements of the presented research. Variables used for the segmentation helped to highlight the internal significant differentiation of parents of 6–8 year-olds in their pro-environmental attitudes and awareness. Different terms can be used to describe consumers engaged in sustainable consumption behaviors. “Green consumption” is related to environmentally responsible consumers who consider the environmental impact of purchasing, using, and disposing of various products, and their use of various green services [57]. Consumers who prefer products or services which do least damage to the environment as well as those which support forms of social justice are often referred to as “green”, “environmental”, “ethical”, “responsible” or “sustainable” consumers. In contrast the term “grey consumer” is used for consumers who generally do not have green values or lifestyles [58]. In our study CL1 and CL2 totaling 73% of the sample described factors linked to sustainability as important when purchasing food for children. However CL2 (29% of the total sample) was found to be the more engaged in raising conversations and teaching their children about food sustainability practices. This suggests that CL2 is potentially the most “green” group in the sample. However almost all of the pre-specified factors influencing food choices were important to members of CL2, pointing to the fact that they are possibly “open” to almost all influences, not necessarily pro-environmental ones. In this neutral, absorbent, open, “pale-green” cluster the number of children in the household was statistically lower and the income was higher compared to the other segments. In contrast the least engaged—“grey” cluster, constituting 27% of the total sample ranked “individual” factors (incl. price and convenience) higher than the “sustainable” factors. Interestingly, during the transition from unwillingness to act to performing the desired behavior, people might be motivated by different benefits associated with the new behavior. Knowing which motives encourage consumers to adopt ecological food consumption patterns could be useful for future campaigns promoting these behaviors [30]. In the case of Polish urban consumers price incentives and opinions of health specialists would potentially support “greener” consumption behaviors among the currently least engaged parents.

Early school years are an important period for building a strong foundation for children to become creative, capable, independent, responsible, and resilient individuals. United Nations named 2005–2014 the Decade of Education for Sustainable Development and sought to mobilize the educational resources of the world to help create a more sustainable future. It has also been stated that one of the paths to sustainability is education and without learning sustainable development cannot be achieved [58,59,60]. Therefore, children should be introduced early on to the linkages between the environment, animals, plants, and human health, as well as the behaviors that they can adopt and action that they can take to protect their health and respect nature [61,62]. Our research showed however that the most frequent reason for not including the child in conversations about sustainable food consumption among Polish caregivers was the child’s age (6–8 years). Recent research also confirmed that environmental messages linked to food might not be understood by children under the age of 10 [63], so despite the fact that it is possible to introduce some chosen subjects to small children—one Australian study found that parents initiate conversations about the source of meat in their diets with children as young as under the age of five [64]—our results suggest that it may be best to focus on slightly older children when raising awareness on more complex sustainable food consumption issues behaviors. The optimal age may be 8–11 years as parents have the greatest influence on their children under the age of twelve and once adolescence starts children become increasingly independent and look at their peers for guidance.

Limiting plastic bottle water purchases was one of the least frequently discussed topics and differed significantly between clusters. In the last decade several high-budget educational campaigns directed to young children were launched in Poland addressing the issue of drinking water for health [65]. While drinking water instead of sugar-sweetened beverages is undoubtedly beneficial from the health point of view, such a campaign may also build the belief that bottled water is the best alternative. A different message is communicated by local authorities, which supply good quality tap water to households and promote reusables bottles [38]. Although currently consumption of water in plastic bottles in Poland is below the European Union’s average [66] from the environmental point of view it is important to teach children to limit consumption of all food and beverages packed in plastic. In 2014, about 98% of the population had access to water from municipal water supplies with a quality in accordance with the requirements specified in the decision of the Minister of Health on potable water quality [67].

Reducing consumption of animal products such as meat or eggs was discussed with children in less than half of the surveyed households. These last issues require ecological awareness and wider perspective on the impact of humans on the natural environment. Awareness about the link between meat production and the degradation of the natural environment remains still on a very low level in Western incl. European Union populations [68]. There is a lack of data concerning this issue in Poland. However it is stated that a diet with lower or even no meat is suitable for children on every stage of development [69,70]. Marketing of highly processed foods makes healthy choices difficult for parents with limited nutritional knowledge. In our sample this knowledge was rather high as around 70% of the interviewed adults confirmed discussing the need to increase consumption of natural, minimally processed foods as well as reducing the consumption of highly processed foods.

In general the surveyed respondents living in urban Poland were engaged in raising their children’s awareness on the links between food, environment and health. 70% declared teaching their child to pay attention to the natural environment in the context of producing and consuming food and 80% confirmed that the responsibility for teaching children lies with parents. Other studies [71,72] showed that schools, internet and television are the main sources of environmental information and can shape sustainable food behaviors of children and adolescents. However parents have a significant impact on their children’s knowledge of and attitudes towards the environment also [41]. The COVID-19 pandemic further highlights the urgent need for heightened awareness of the inextricable links between healthy people, healthy societies and a healthy planet. Empowered parents together with the global framework of learning, including primary and secondary schools and NGOs, should support communities in taking steps to improve planetary health. This will help prepare the young generation for the challenges ahead and nurture them into future agents of change, advocating for a healthier and more sustainable way of living.

A holistic approach to environmental education involving parents seems to be under development only in recent years [61,62]. This corresponds with an insufficient knowledge of parents in the field of sustainable behaviors however, building the pro-environmental habits requires changes in a whole food system design to encourage them. One of the local nudges are school gardens, which improve parents’ nutritional and agricultural knowledge and increase children’s liking for vegetables [73]. 

Dynamic processes and influences during a person lifetime shape food choices and collective changes in consumer behavior can open a pathway towards more sustainable food systems [74]. Such systems are being increasingly challenged to provide adequate, safe, diversified and nutrient rich food that contributes to healthy diets due to constraints posed by resource scarcity, environmental degradation as well as by unsustainable production and consumption patterns. Considering the concerns raised about their sustainability, there is an urgent need to promote diets that are healthy and have a low environmental impact. Our study found that only 27% of respondents indicated the correct meaning of the term “sustainable food consumption”. However it must be noted that this result was higher than in other studies conducted in Poland among city dwellers [42,43]. In comparison the share of respondents who were familiar with this term in Polish rural households was only 9% [75]. One of the potential limitations of the study is that although food sustainability has three dimensions: economic, environmental and social (also described as the profits, planet, and people pillars) our research focuses mainly on the environmental perspective. Even so, although many sustainability-related issues were included in the questionnaire their final list had to be reduced otherwise it would be overwhelming and confusing for respondents. Another limitation of the study arises from the attitude-behavior gap which is typical for consumer studies. Despite the fact that the survey was conducted anonymously, the answers could be influenced by the matter of the research itself. It can be assumed that as parents’ knowledge about sustainable food patterns and behaviors is limited, it entails a narrow spectrum of topics raised in conversations with children at home. 

Another potential limitation is also the rather small number of studies on sustainable food choices carried out in Poland. Hopefully this will change in the next years as initiatives such as the 2021 UN Food System Summit aim to launch actions to transform the way the world produces and consumes food and deliver progress on all 17 Sustainable Development Goals. One of the key steps to this will be initiating dialogue at country levels and supporting more research in this field [76]. In many countries, including Poland further research directions should include exploration of changes in food sustainability awareness among different population groups—incl. families living in rural areas. Additionally the impact of COVID-19 disruptions of school education should be analysed in the context of the role of family members (including parents) in teaching children about the links between food, health and the environment. Following the UN Food Systems Summit in 2021 research should also be conducted among policy-makers and other participants of the food chain, such as food producers and NGOs, to evaluate their engagement in the process of shifting food behaviors towards sustainability.

## 5. Conclusions

The consequences of unhealthy dietary behaviors at population level are worrying not only from the health, but also social, economic and environmental perspectives. Parental food choices exert a significant influence on the diets of their children but at the same time children’s preferences impact their caregivers’ purchasing decisions. Starting in early years, children and their guardians need to be active participants in planetary health education and shifting family diets and food choices towards sustainability. Building children’s awareness and preparing them to deal with the complex nature of sustainability in their future needs new, research-based holistic approaches. The development of specific child-centered tools (for example interactive board games) targeted at 8–11 year-olds could further enable and effectively support parental engagement in discussions on the links between food, environment and health. Families should also be encouraged to take part in out-door activities aimed at promoting sustainable food practices like green fairs, farmers’ markets and urban gardens and to volunteer in initiatives reducing food waste and buying seasonal products. Sustainability should also be incorporated in public health, food and nutrition messages and policies, incl. food based dietary guidelines, especially those aimed at children. The presented data indicates that Polish parents feel responsible for teaching their children and therefore information and awareness-raising programmes on these issues would help them feel more empowered to do so. 

## Figures and Tables

**Table 1:** Socio-demographic characteristics of respondents and their child.

Adult Respondent	n (% of N)	Child	n (% of N)
Gender		Gender	
Female	701 (68)	Girl	539 (52)
Male	334 (32)	Boy	496 (48)
Age (years)		Age (years)	
20–29	82 (8)	6	373 (36)
30–39	725 (70)	7	357 (34)
40–49	198 (19)	8	305 (30)
50+	8 (1)		
No data	22 (2)		
Education		Type of school	
Primary or vocational	70 (7)	Public elementary	925 (89)
Secondary	384 (37)	Private elementary	41 (4)
Higher	581 (56)	Preschool	69 (7)
Number of children in the household		Child’s body weight	
1		Normal	
2	324 (31)	Overweight	866 (84)
3	569 (55)	Underweight	91 (9)
4 and more	108 (10)		78 (7)
	34 (4)		
Household’s income		Child’s diet ^1^	
(PLN ^2^ per person per month)		Typical	677 (65)
Up to 1500		Gluten free	30 (3)
1501–3000	367 (35)	No meat	13 (1)
3001 and more	327 (32)	No animal products	10 (1)
Denial	204 (20)	No/limited sweets	
	137 (13)	No/limited dairy	162 (16)
		Low in carbohydrates	73 (16)
		Other	117 (11)
			34 (3)

^1^ Possibility to choose more than one answer; ^2^ BSc, MSc or higher degree. PLN—Polish Złoty is the official currency and legal tender of Poland.

**Table 2:** Post-hoc analysis of the total sample and cluster means for factors determining parental food choices.

Factors	Total Sample N = 1035	Clusters		
		CL1 n = 458	CL2 n =304	CL3 n = 273
**I. Universal**				
Child’s health	4.63	4.89 ^a^	4.58 ^b^	4.25 ^c^
Taste	4.47	4.62 ^a^	4.54 ^a^	4.12 ^b^
Principles of healthy eating	4.26	4.45 ^a^	4.51 ^a^	3.67 ^b^
**II. Sustainable (collective/we)**				
Symbols certifying the product’s special-quality attributes	4.12	4.32 ^a^	4.48 ^a^	3.38 ^b^
Local origin to reduce food transport	3.91	4.10 ^b^	4.42 ^a^	3.02 ^c^
Organic production	3.89	4.07 ^b^	4.42 ^a^	2.99 ^c^
Protection of the natural environment	3.77	3.89 ^b^	4.37 ^a^	2.89 ^c^
Reusable packaging	3.56	3.57 ^b^	4.29 ^a^	2.72 ^c^
**III. Individual (me/my household)**				
Local origin to support Polish producers	3.95	4.13 ^b^	4.40 ^a^	3.15 ^c^
Price	3.92	3.80 ^b^	4.38 ^a^	3.62 ^b^
Certified high quality of product	3.82	3.99 ^b^	4.41 ^a^	2.87 ^c^
Convenience	3.80	3.68 ^b^	4.39 ^a^	3.36 ^c^
**IV. External (opinions of others/they)**				
Recommendations of health professionals	3.99	4.16 ^b^	4.41 ^a^	3.26 ^c^
Recommendations of research institutes or experts	3.61	3.64 ^b^	4.35 ^a^	2.72 ^c^
Teachers’ opinions	3.21	2.98 ^b^	4.17 ^a^	2.52 ^c^
Opinions of the child’s peers	3.07	2.67 ^b^	4.12 ^a^	2.57 ^b^
Advertising	2.72	2.13 ^b^	3.93 ^a^	2.35 ^b^
Consumer trends	2.60	2.01 ^b^	3.88 ^a^	2.17 ^b^

Superscript letters ^a^, ^b^, ^c^ indicate if there are significant clusters differences between cluster means of each factor (*p* ≤ 0.001). If two cluster means have the same superscript letter, then they do not differ statistically.

**Table 3:** Socio-demographic characteristics of the studied sample, by clusters.

Characteristics	CL1 n = 458	CL2 n = 304	CL3 n = 273
**Gender (%)**			
Women	70	69	64
Men	30	31	36
**Age**			
Mean (SD)	36 (5)	36 (4)	36 (5)
**Education (%)**			
Primary or vocational	6	5	9
High school	36	35	41
Bachelor’s degree and higher	58	60	50
**Children under age of 18 ^1^**			
Median	2.0	1.0	2.0
Mean (SD)	2.0 (0.8)	1.6 (0.8)	1.95 (0.7)
**Children’s age (%)**			
6	33	22	31
7	33	45	25
8	34	33	44
**Type of school (%) ^1^**			
Public elementary	91	92	84
Private elementary	3	3	6
Preschool	6	5	10
**Household’s income (%) ^1^** **(PLN per person per month)**			
Up to 1500	50	24	44
1501–3000	32	47	32
3001 and more	18	29	24

^1^ Indicate significantly different means between clusters following ANOVA tests at *p* ≤ 0.001.

**Table 4:** Topics raised by parents with their 6–8 year-old children (%).

Topic	Total Sample N = 1035	Clusters		
		1 n = 458	2 n = 304	3 n = 273
Maintaining health with proper nutrition	90	98 ^a^	92 ^a^	73 ^b^
Sorting of waste	89	95 ^a^	92 ^a^	74 ^b^
Limiting food waste	88	95 ^a^	90 ^a^	73 ^b^
Sorting of food packaging	86	93 ^a^	90 ^a^	68 ^b^
Buying and eating seasonal food	83	88^a^	92^a^	64^b^
Necessity to protect the natural environment	79	86 ^a^	91^a^	54 ^b^
Hunger in Poland and worldwide	72	76 ^a^	87 ^a^	49 ^b^
Increasing consumption of natural foods	69	76 ^a^	83 ^a^	43 ^b^
Reducing the consumption of highly-processed foods	69	74 ^a^	85 ^a^	44 ^b^
Buying locally-produced foods in order to limit transport	58	58 ^b^	84 ^a^	28 ^c^
Limiting plastic bottle water purchases	44	38 ^b^	76 ^a^	19 ^c^
Reducing consumption of animal products, i.e., meat or eggs	39	31 ^b^	70 ^a^	18^c^

Superscript letters indicate if clusters differ significantly for each factor (*p* ≤ 0.001).

## Data Availability

The data presented in this study are available on request from the corresponding authors.

## Supplementary Materials

The following are available online at https://www.mdpi.com/1660-4601/18/6/3225/s1, Table S1. Survey questions analyzed in the paper.
