# Supplementary material for: Parental Food Choices and Engagement in Raising Children’s Awareness of Sustainable Behaviors in Urban Poland
Source: Int J Environ Res Public Health. 2021 Mar 20;18(6):3225. doi: 10.3390/ijerph18063225 (PMC8003873; doi:10.3390/ijerph18063225)
Supplement: Supplementary file 1 [file ijerph-18-03225-s001.pdf]

Supplementary files:

Table S1. Survey questions analysed in the paper

Question	Answers/options
Q1. Please name the most important factors determining your choice when buying food for your child<br>*Open-ended question, max. three factors*	1.<br>2.<br>3.
Q2. Please indicate to what extent each of the factors (randomly rotated) is important to you when choosing food for your child<br>*Single-response matrix question with a five-level Likert-type scale: very important // rather important // neither important nor unimportant// rather unimportant // unimportant*	Price<br>Child's health<br>Protection of the natural environment<br>Taste<br>Organic production<br>Opinions of the child's peers<br>Convenience (food easy to prepare)<br>Reusable packaging<br>Certified high quality of product<br>Local origin to support Polish producers<br>Local origin of to reduce food transport<br>Principles of healthy eating<br>Symbols certifying the product's special-quality attributes<br>Advertising<br>Consumer trends<br>Recommendations of health professionals<br>Recommendations of research institutes or experts<br>Teachers' opinions
Q3. Do you discuss the following topics in the presence of your child?<br>*Dichotomous question*<br>*Yes/No*<br>*Topics randomly rotated*	Buying / eating seasonal food<br>Reducing food waste<br>Reducing consumption of animal products i.e. meat or eggs<br>Buying local products to reduce food transport<br>Increasing consumption of natural, minimally-processed food<br>Limiting plastic bottle water purchases<br>Reducing consumption of highly processed products<br>Waste segregation<br>Sorting food packaging<br>Need to protect the natural environment<br>Prevalence of hunger in Poland and worldwide<br>Maintaining health with proper nutrition

Q4. Why do you not include your child in conversations on the following topics?<br>*Single choice matrix question*<br><br>*Topics – as in Q3*	I don't know enough about it<br>I'm not interested in it<br>I don't have time<br>I think it is not an important problem<br>The child is still too young<br>The school should take care of it<br>Other reason
Q5. Are you familiar with the term "sustainable food consumption"?<br>*Dichotomous question*	Yes<br>No
Q6. What do you think is meant by "sustainable food consumption"?<br>*Single choice question*	The energy value of consumed food equals the body's energy expenditure<br>The share of plant and animal products in the food consumed is the same<br>Everyday diet is carried out so as to minimize the influence on the natural environment<br>The cost of nutrition is adapted to the financial capabilities of the household<br>Other (please enter)
Q7. Do you teach your child to pay attention to the natural environment in the context of producing and consuming food?<br>*Single choice question*	Yes, I have been doing this for years<br>Yes, only recently<br>No, but I'm going to do it soon<br>No and I'm not going to do it because…
Q8. Who is responsible for teaching children about the following topics?<br>*Multi-response question:*<br>*Parents / Grandparents and other adult family members / School / Media (TV, internet, press) / Others*	A. Environmental protection<br>B. The link between diet and health<br>C. Avoiding food waste
